# A prospective comparison of adaptive and fixed boost plans in radiotherapy for glioblastoma

**DOI:** 10.1186/s13014-022-02007-4

**Published:** 2022-02-22

**Authors:** Tomohiko Matsuyama, Yoshiyuki Fukugawa, Junichiro Kuroda, Ryo Toya, Takahiro Watakabe, Tadashi Matsumoto, Natsuo Oya

**Affiliations:** 1 Department of Radiation Oncology, Kumamoto University Hospital, 1-1-1, Honjo, Chuo-ku, Kumamoto-shi, Kumamoto 860-8556 Japan; 2 Department of Neurosurgery, Kumamoto University Hospital, Kumamoto, Japan

**Keywords:** Glioblastoma, Adaptive radiotherapy

## Abstract

**Purpose:**

To analyze the efficacy of adaptive radiotherapy (ART) for glioblastoma.

**Methods:**

Sixty-one glioblastoma patients who received ART were prospectively evaluated. The initial clinical target volume (CTVinitial) was represented by T2 hyperintensity on postoperative MRIs (pre-RT MRI [MRIpre])plus 10 mm. The initial planning target volume (PTVinitial) was the CTVinitial plus a 5-mm margin. The PTVinitial received 40 Gy. An MRI and a second planning CT were performed during radiotherapy (MRImid). Two types of boost CTVs (the resection cavity and residual tumor on enhanced T1-weighted MRI plus 10 mm) were created based on the MRIpre and MRImid (CTVboost-pre and -mid). The boost PTV (PTVboost) was the CTVboost plus 5 mm. Two types of boost plans (fixed and adaptive boost plans in the first and second planning CT, respectively) of 20 Gy were created. The PTV based on the post-RT MRI (PTVboost-post) was created, and the dose-volume histograms of the PTVboost-post in the fixed and adaptive boost plans were compared. Additionally, the conformity indices (CIs) of the fixed and adaptive boost plans were compared.

**Results:**

The median V95 of the PTVboost-post of the fixed and adaptive boost plans (V95pre and V95mid) were 95.6% and 98.3%, respectively (*P* < 0.01). The median V95pre and V95mid of patients after gross total resection (GTR) were 97.4% and 98.8%, respectively (*P* = 0.41); in contrast, the median values of patients after non-GTR were 91.9% and 98.2%, respectively (*P* < 0.01). The median CIs of the fixed and adaptive boost plans in all patients were 1.45 and 1.47, respectively (*P* = 0.31). The median CIs of the fixed and adaptive boost plans in patients after GTR were 1.61 and 1.48, respectively (*P* = 0.01); in contrast, those in patients after non-GTR were 1.36 and 1.44, respectively (*P* = 0.13).

**Conclusion:**

ART for glioblastoma improved the target coverage and dose reduction for the normal brain. By analyzing the results according to the resection rate, we can expect a decrease in normal brain dose in patients with GTR and an increase in coverage in those with partial resection or biopsy.

## Background

The current standard therapy for glioblastoma is maximal surgical resection followed by postoperative radiation therapy (RT) of 60 Gy delivered in 30 fractions combined with temozolomide [1]. While postoperative RT prolongs survival in patients with glioblastoma, this tumor is highly refractory; therefore, the size of the tumor may increase during the treatment period because of its resistance to treatment [2, 3].

Previous studies have reported changes in the target during postoperative RT for glioblastoma [2–8]. For cases in which the target volume increased during the irradiation period, continuing treatment according to the initial RT plan may result in a decrease of the target volume coverage. In contrast, the resection cavity may decrease during chemoradiotherapy. If the resection cavity decreases, a large volume of the normal brain may receive a significant dose of radiation.

Adaptive radiation therapy (ART) is a treatment technique that corrects RT plans according to structural changes in the tumor and normal tissue during the treatment period. In the above scenarios, ART could improve the dose coverage of the target and reduce radiation delivered to the normal brain tissue. However, the application of ART for glioblastoma has not been sufficiently examined.

The purpose of this study was to prospectively evaluate the efficacy of ART for glioblastoma by comparing adaptive boost and fixed boost (based on pre-RT MRI) methods.

## Methods

### Study population

In this clinical trial, we prospectively evaluated patients with newly diagnosed glioblastoma who received postoperative ART of 60 Gy in 30 fractions using the protocol described below. Eighty-seven patients diagnosed with glioblastoma received RT at our institution from March 2015 to October 2018. Of the 87 patients, 17 received short-term irradiation of 40.5 Gy due to old age or frail condition, and 2 did not participate in this study because of special target setting in another clinical trial. Seven patients were treated with intensity-modulated RT (IMRT); however, for reasons described below, those treated with IMRT were not included. As a result, 61 of 87 patients were included in the study.

All patients underwent surgery or biopsy and were pathologically confirmed to have glioblastoma. This prospective study was approved by the institutional review board of Kumamoto University Hospital (IRB number 1893), and all participants provided written informed consent. All patients were treated with temozolomide during and after RT.

### Treatment planning and adaptive RT

A postoperative, pre-RT, MRI (MRIpre) was performed within 3 days of resection in all patients. MRI scans were acquired using a Siemens 3 T Magnetom Prisma fit (Siemens, Erlangen, Germany). T2weighted images (FOV = 230 × 230 mm, TE = 90.6 ms, TR = 4500 ms) were acquired with a slice thickness of 5 mm, and a 371 × 512 matrix. 3DT1 weighted images (FOV = 230 × 230 mm, TE = 3.63 ms, TR = 2200 ms, FA = 9.0°) were acquired with intravenous gadolinium contrast (gadopentetate dimeglumine:Magnevist; Bayer Yakuhin, Osaka, Japan) using a reconstructed slice thickness of 1.1 mm, and a 187 × 256 matrix. The extent of tumor removal was classified as gross total resection (GTR), partial resection (PR), or biopsy based on neurosurgeon’s and neuroradiologist’s assessments.

In this study, patients were treated with 3- or 4-field 3D conformal RT (3DCRT). For postoperative RT, the first planning simulation computed tomography (CTpre) was performed 2 to 3 weeks after surgery. The patients were immobilized using a tight thermoplastic mask. Non-contrast medium-enhanced CT images were acquired on a GE LightSpeed CT simulator (GE Healthcare, Chalfont St. Giles, UK), and 1.0 mm slices were obtained. RT was planned using Eclipse version 13 (Varian Medical Systems, PaloAlto, CA, U.S.). The CTpre images were fused with the thin-slice MRIpre images using image-fusion software. The initial clinical target volume (CTVinitial) was defined as a hyperintense signal abnormality on the T2 weighted MRIpre (including the residual enhanced tumor and resection cavity on the postoperative contrast-enhanced T1 weighted MRI) plus a 1 cm margin containing normal brain tissue with consideration of barriers including as bones and dura. The initial planning target volume (PTVinitial), which was created by adding a 0.5-cm margin to the CTVinitial, received 40 Gy at the isocenter in 20 fractions (initial plan).

**Fig. 1:**
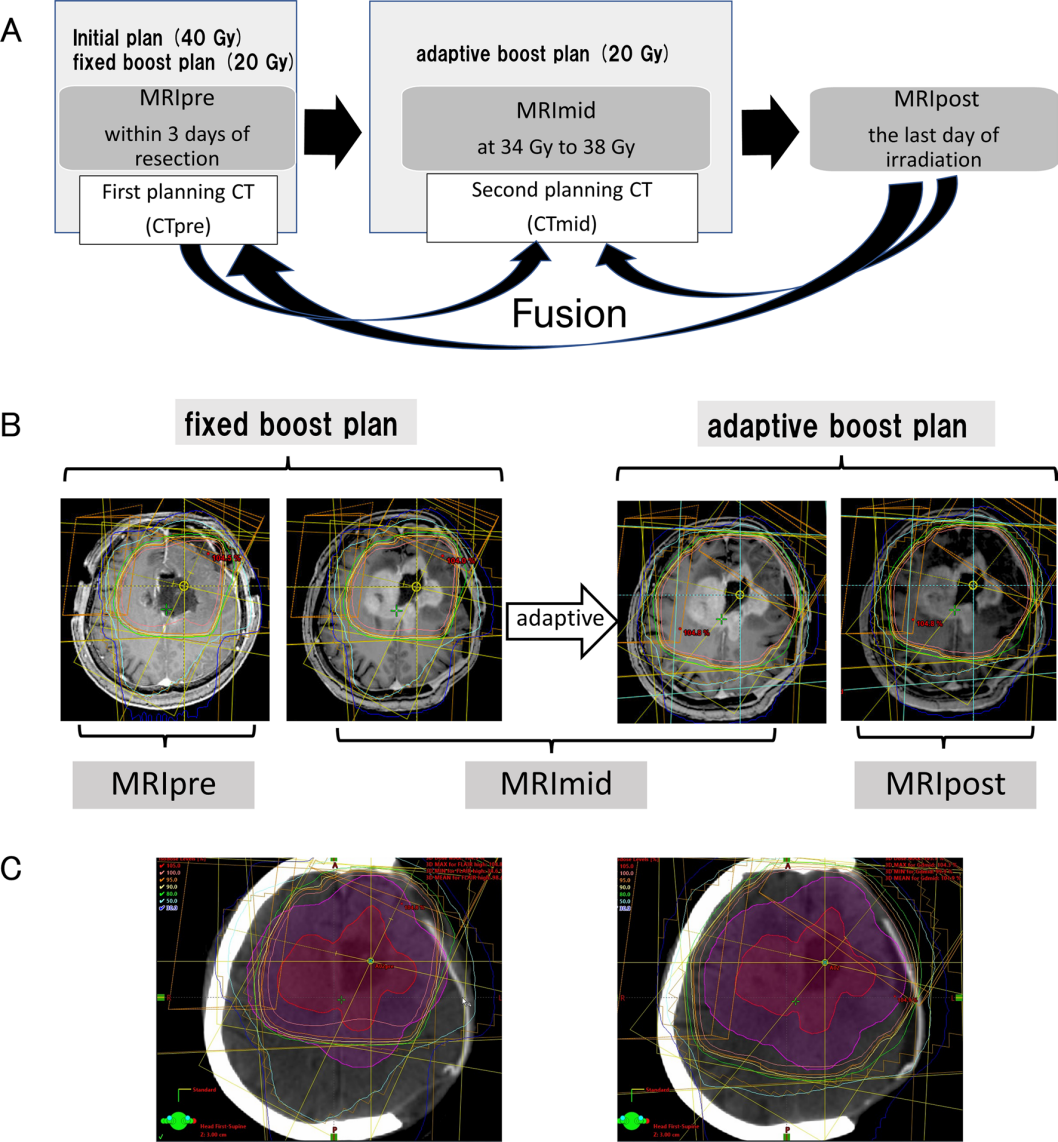
**A** CT and MRI procedures for ART. The fixed boost plan and adaptive boost plan are created on CTpre and CTmid, respectively. The dose distributions of PTVpost (PTV created by MRIpost) in the fixed boost plan and adaptive boost plan are compared by fusing MRIpost to CTpre and CTmid. **B** MRI with three timings (Pre, Mid, Post) and two boost plans. **C** Left: GTV-post (red) and PTVboost-post (magenta) on the CTpre (fixed boost plan). Right: GTV-post (red) and PTVboost-post (magenta) on the CTmid (adaptive boost plan). ART, adaptive radiation therapy; CT, compute tomography; GTV, gross target volume; MRI, magnetic resonance imaging; PTV, planning target volume

For ART, a contrast-enhanced MRI during RT (MRImid) was performed at an irradiation dose of 34–38 Gy. The second planning CT (CTmid) was performed on the same day as the MRImid, and images were fused with those from the MRImid. The CT and MRI procedures used in this study are shown in Fig. 1A.

**Fig. 2:**
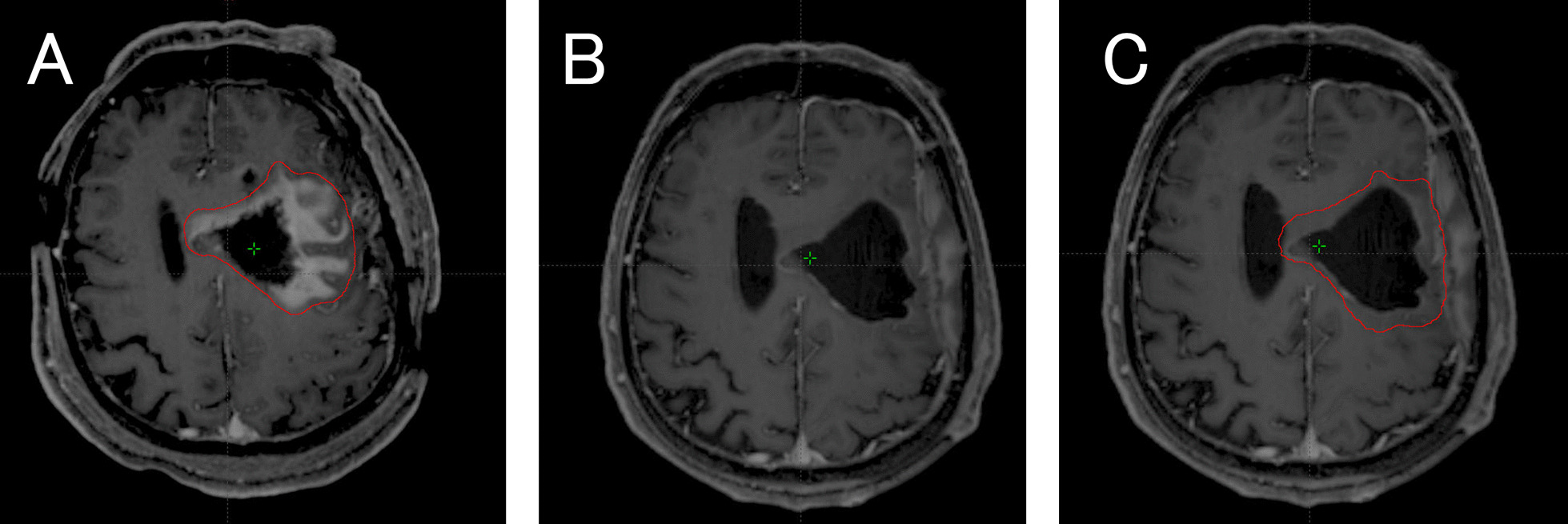
Contouring procedures in ART. **A** Boost GTV (GTV-pre) in postoperative contrast-enhanced T1 weighted MRI (MRIpre). **B** The contrast enhancement area was decreased in contrast-enhanced MRI during radiotherapy (MRImid). **C** Boost GTV (GTV-mid) in MRImid. ART, adaptive radiation therapy; GTV, gross target volume; MRI, magnetic resonance imaging

In this study, two types of boost gross target volumes (GTV-pre and GTV-mid) were created based on the MRIpre and MRImid, respectively. GTV-pre was defined as the residual enhanced tumor and resection cavity on the contrast-enhanced T1 weighted MRIpre. GTV-mid was created as follows: when the contrast-enhanced area was expanded on the MRImid, GTV-mid was modified according to the expansion. If the contrast-enhanced area shrank or disappeared on the MRImid, the brain tissue in the originally enhanced area of the MRIpre was included in the GTV-mid (Fig. 2). The GTV-mid was modified according to changes in the surgical cavity and mass effect. The GTV-pre and GTV-mid were contoured to the CTpre and CTmid, respectively. Two types of boost clinical target volumes (CTVboost-pre and CTVboost-mid) were represented by GTV-pre plus 1.0 cm and GTV-mid plus 1.0 cm, respectively. The two types of boost planning target volumes, PTVboost-pre and PTVboost-mid, were CTVboost-pre and CTVboost-mid plus 0.5 cm, respectively.

The eyeballs, lens, optic nerves, optic chiasma, brain stem, and normal brain were delineated as organs at risk (OAR) on the CTpre and CTmid. Two types of boost plans (pre-boost plan [fixed boost plan] in the CTpre and mid-boost plan [adaptive boost plan] in the CTmid) of 20 Gy in 10 fractions were created (Fig. 1B). In the actual RT, using the adaptive boost plan, the PTVboost-mid received 20 Gy in 10 fractions at the isocenter. To achieve clinically safe treatment with regards to the OARs, the fixed boost plan was created to satisfy the dose constraint in the sumplan, the sum of the initial plan and the fixed boost plan. The adaptive boost plan was created so that the OAR dose was not higher than that of the fixed boost plan. When the OAR dose in the adaptive boost plan exceeded that of the fixed boost plan due to unavoidable reasons such as tumor growth, the OAR dose was evaluated by creating a sumplan of the initial plan and the adaptive boost plan by fusing the CTmid images with the CTpre images. In some cases, the fixed boost plan was the conventional RT, but the adaptive boost required the IMRT technique to reduce the dose of OARs. Because the comparison between conventional and IMRT plans might have impacted results [9–11], we excluded cases treated with IMRT in this study.

### Data analysis

In this study, we evaluated the efficacy of ART by comparing the coverage of two boost plans for the boost PTV (PTVboost-post), which was created based on post-RT MRI (MRIpost). The MRIpost was performed on the last day of irradiation, and the CTVboost-post was created based on the MRIpost in the same way as the CTVboost-mid. The PTVboost-post was created by adding a 0.5-cm margin to the CTVboost-post. To assess the effectiveness of ART for glioblastoma, the MRIpost images were fused with the CTpre and Ctmid images (Fig. 1C). A dose-volume histogram (DVH) of the PTVboost-post in the fixed and adaptive boost plans were evaluated using the percent volume that received at least 95% and 90% of the prescription dose (V95 and V90, respectively). The PTV coverage evaluation was performed in all cases considering the extent of tumor resection (GTR, PR, and biopsy only). Additionally, the conformity indices (CIs) of the fixed and adaptive boost plans were compared. In this study, CI was defined as the ratio of the volume that received 95% of the prescribed dose to the PTV [12] (RTOG conformity index). To assess the dose close to the maximum dose (D2%) to the OAR, DVHs of OARs based on MRIpost were generated and compared for both the fixed and adaptive boost plans. Values of the relative volume of normal brain tissue receiving at least nGy (Vn [%]) in the fixed boost plan were compared to those in the adaptive boost plan using the DVHs for normal brain tissue. The normal brain was defined as the volume of the whole brain minus the PTVboost-post.

A Wilcoxon signed-rank test was performed to compare the coverage of the PTV-post, CI, and OAR doses in the two plans. Statistical analyses were performed using the Stata Statistical package version 13, and differences of *P* < 0.05 were considered statistically significant.

## Results

All 61 patients included in this study were treated with temozolomide during ART. The median age was 64 years (range 4–78 years). GTR was achieved in 31 patients, and PR was achieved in 23; the remaining 7 underwent biopsy only. Table 1 summarizes patient characteristics. One patient had 122 days between the initial surgery and the start of RT. This patient refused additional treatment after the surgery and was followed up with anticonvulsants only, but recurred and developed vertigo, therefore he received chemoradiation therapy. In this patient, the MRI at the time of recurrence was used as MRIpre for treatment planning.

**Table 1:** Patient characteristics (n = 61)

Variable	n (%)
Sex	
Male	36 (59)
Female	25 (41)
Age (median, range)	64,4–78
Extent of surgical resection	
GTR	31 (51)
PR	23 (38)
biopsy	7 (11)
Location of tumor	
Frontal	17 (28)
Parietal	13 (21)
Temporal	22 (36)
Occipital	3 (5)
Thalamus	2 (3)
Cerebellum	2 (3)
Multi focal	2 (3)
O6-methylguanine-DNA methyl-transferase status	
Methylated	30 (49)
Unmethylated	21 (34)
Unknown	10 (16)
Days from surgery to radiation (median, range)	18 (9–122)

In 36 of the 61 patients, expansion of the contrast-enhanced area on the MRImid (excluding the linear enhancement along the resection cavity wall that was determined to be postoperative change) compared to that on the MRIpre was observed. Seventeen of the 36 patients with an expansion of the contrast-enhanced area on the MRImid had GTR, while the remaining 19 underwent PR or biopsy only. In four cases, the MRImid enhancement tumor appeared newly within 5 mm of the edge of the irradiation field in the fixed boost plan, and two of these cases had a deviation from the irradiation field.

The median volumes of the GTV-pre and the GTV-mid were 35.3 mL (range 2.9–158.9 mL) and 35.3 mL (range 2.6–283.9 mL), respectively, and the mean ratio of the GTV-pre to the GTV-mid volumes (GTV-mid/GTV-pre) was 1.11. The mean ratio of the GTV-pre to the GTV-mid volumes in patients with and without GTRwere 0.84 and 1.30, respectively (*P* < 0.01).

### Comparisons of DVH and CI between the fixed and adaptive boost plans

**Fig. 3:**
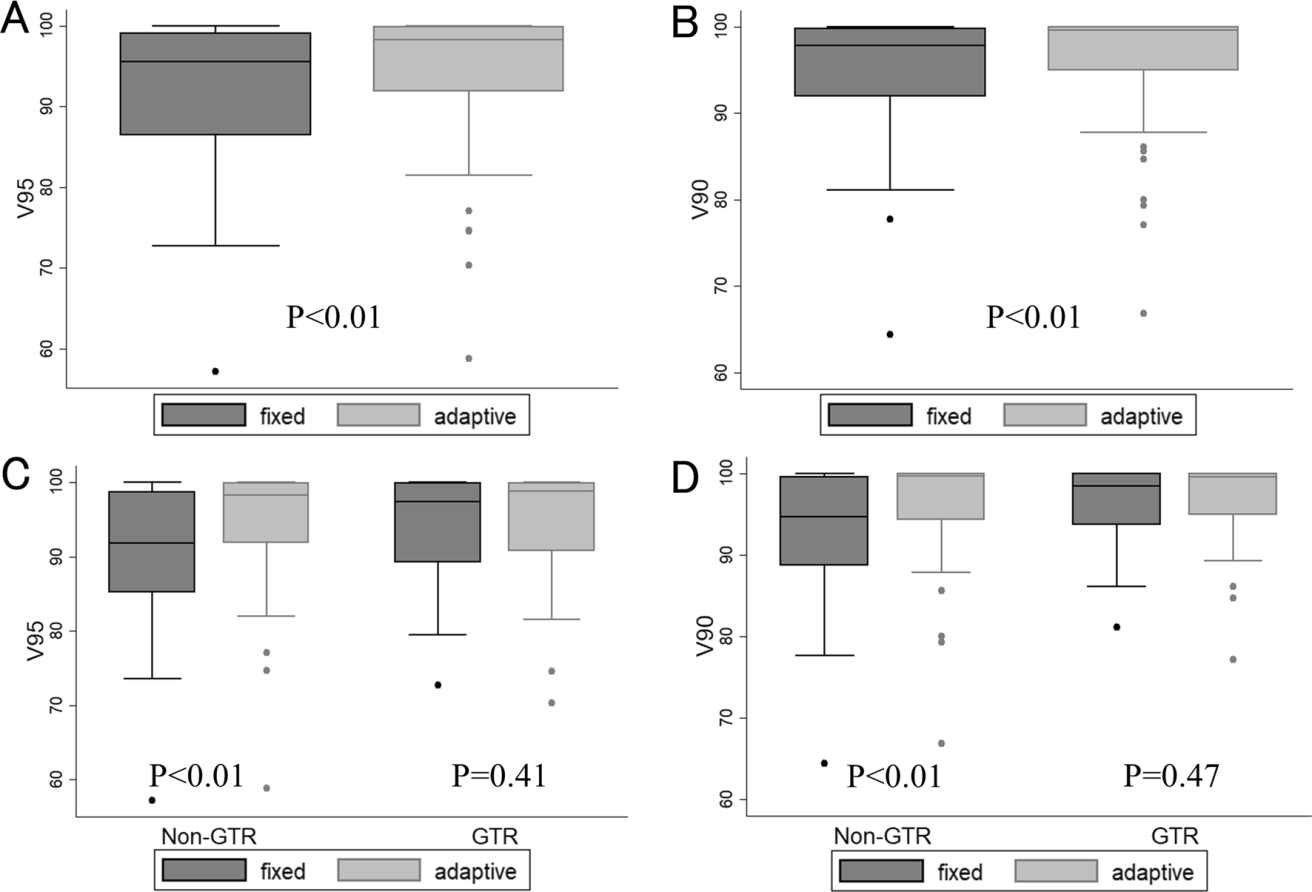
V95 and V90 of PTV-post in the fixed boost plan and adaptive boost plan. **A** V95 in all patients **B** V90 in all patients **C** V95 according to resection rate **D** V90 according to resection rate. GTR, gross total resection; PTV, planning target volume

In all patients, the median V95 of the PTVboost-post in the fixed boost plan (V95pre) and the adaptive boost plan (V95mid) were 95.6% (range 57.2–100%) and 98.3% (range 58.8–100%), respectively, and median V90pre and V90mid values were 97.9% (range 64.5–100%) and 99.6% (range 66.7–100%), respectively (*P* < 0.01 and *P* < 0.01, respectively) (Fig. 3A, B). The median V95pre and V95mid values in patients with GTR were 97.4% (range 72.7–100%) and 98.8% (range 70.3–100%), respectively (*P* = 0.41), and the median V90pre and V90mid values in patients with GTR were 98.5% (range 81.1–100%) and 99.6% (range 77.1–100%), respectively (*P* = 0.47). In contrast, the median V95pre and V95mid values in patients withPR or biopsy were 91.9% (range 57.2–100%) and 98.2% (range 58.8–100%), (*P* < 0.01). In a similar fashion, V90pre (median 94.7%, range 64.4–100%) was significantly lower than V90mid (median 99.7%, range 66.8–100%) in patients who underwent PR or biopsy (*P* < 0.01) (Fig. 3C, D).

**Fig. 4:**
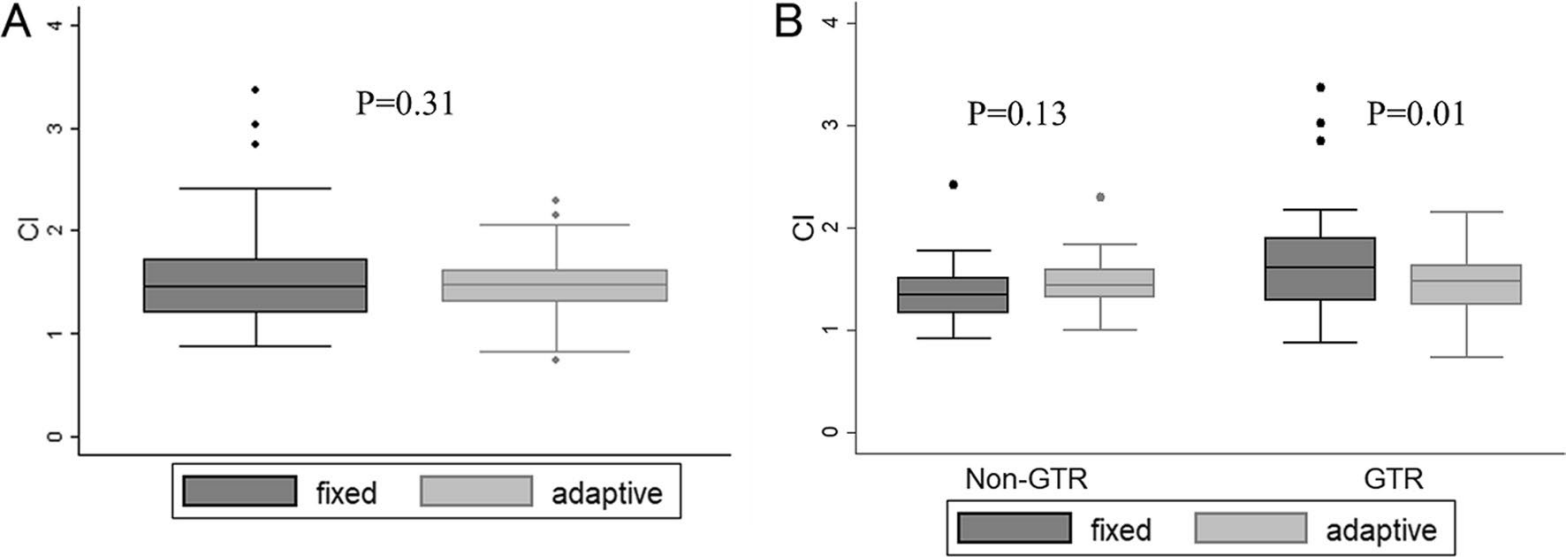
Conformity index (CI) of fixed boost plan and adaptive boost plan. **A** CI in all patients **B** CI according to resection rate. GTR, gross total resection

Median CIs of the fixed boost plan and adaptive boost plan (CIpre and CImid) in all patients were 1.45 (range 0.87–3.37) and 1.47 (range 0.73–2.30), respectively (*P* = 0.31) (Fig. 4A). CIpre and CImid in the 31 patients with gross total resection were 1.61 (range 0.87–3.37) and 1.48 (range 0.73–2.15), respectively (*P* = 0.01); in contrast, CIpre and CImid in the 30 patients with partial resection or biopsy were 1.36 (range 0.92–2.42) and 1.44 (range 1.00–2.30), respectively (*P* = 0.13) (Fig. 4B).

D2% of OARs (optic nerves, optic chiasm, and brainstem) and doses of the normal brain in the fixed boost and adaptive boost plans are shown in Table 2. There was no significant difference in the dose to the normal brain between all patients and those with PR or biopsy, whereas the dose to the normal brain in the adaptive boost plan was significantly lower in patients who underwent GTR. The median V10 of the normal brain for the fixed boost plan and the adaptive boost plan in GTR patients were 28.9 and 26.1 mL (*P* = 0.01), the median V15 were 12.2 and 9.0 mL (*P* = 0.01), and the median V20 were 2.0 and 0.8 mL (*P* = 0.01), respectively.

**Table 2:** Comparison of OARs dose in the Pre-boost plan and Mid-boost plan

	Fixed boost plan	Adaptive boost plan	*P* value
	Median (range)	Median (range)	
D2% to OARs (Gy)			
All patients			
Ipsilateral optic nerve	7.4 (0.3–17.1)	7.3 (0.3–19.9)	0.62
Contralateral optic nerve	5.1 (0.3–12.9)	4.6 (0.3–20.6)	0.98
Optic chiasm	9.2 (0.6–18.9)	9.9 (0.6–20.)	0.22
Brain stem	16.2 (0.9–20.4)	18.2 (0.8–20.4)	0.85
Patients with GTR			
Ipsilateral optic nerve	7.4 (0.3–17.1)	7.6 (0.3–14.6)	0.93
Contralateral optic nerve	5.1(0.3–12.9)	4.6 (0.3–13.5)	0.29
Optic chiasm	8.9 (0.8–17.1)	9.9 (0.6–15.2)	0.20
Brain stem	15.7 (1.7–20.4)	18.5 (0.9–20.4)	0.31
Patients with STR or biopsy			
Ipsilateral optic nerve	7.9 (0.5–15.7)	6.8 (0.5–19.9)	0.59
Contralateral optic nerve	5.2 (0.4–12.4)	4.7 (0.4–20.1)	0.30
Optic chiasm	9.9 (0.6–18.9)	9.9 (0.6–20.2)	0.59
Brain stem	16.9 (0.9–20.)	18.1 (0.8–20.1)	0.40
Vn(%) of normal brain (ml)			
All patients			
V10	35 (3–64.3)	32.5 (9.3–70.7)	0.57
V15	14.2 (22.0–45.9)	14.2 (3.5–46.1)	0.19
V20	2.8 (0–13.2)	2.2 (0–14.8)	0.23
Patients with GTR			
V10	28.9 (11.3–51.7)	26.1 (9.3–56.8)	0.01
V15	12.2 (2.2–23.5)	9 (3.5–30.3)	0.01
V20	2.0 (0–9.4)	0.8 (0–13.7)	0.01
Patients with PR or biopsy			
V10	39.2 (3–64.3)	40.2 (24.1–70.7)	0.17
V15	18.7 (7.4–45.9)	19 (8–46.1)	0.72
V20	3.4 (0.4–13.2)	2.9 (0.3–14.8)	0.59

## Discussion

In this study, it was shown that repeat MRIs and ARTs led to improved coverage of the target and reduced the dose to the normal brain. The PTV coverage was significantly improved in the boost plan based on MRI during the treatment period compared to that based on the postoperative MRI. Analysis by resection rate revealed that the PTV coverage was particularly improved in non-GTR cases; CI and normal brain doses were not different in all cases, whereas ART improved CI and normal brain doses in GTR cases. The GTV for boost plans tended to decrease in patients who underwent GTR and increase in patients who underwent non- GTR (the mean ratio of GTV-mid/GTV-pre was 0.84 and 1.30, respectively).

Several previous studies have reported on the target volume changes during RT for gliomas, includingglioblastoma. Tsien et al. [3] assessed changes in the GTV using MRI during the treatment of glioma patients. They observed a decrease in the GTV in 14 of 19 patients, no change in 2, and an increase in 3. The median decrease in V95 was 10.8% in these 3 patients. Kim et al. [5] evaluated the changes in cavity size in 19 patients with glioblastoma after total resection using CT. A comparison of the cavity size among postoperative patients at initial treatment planning and boost planning showed a median change of 29.0% from postoperative to initial planning, and 34.9% from initial planning to boost planning. The boost plan created with the CT at the time of boost showed a decrease in the high-dose area of the normal brain compared to that created at the time of initial planning. Manon et al. [4] analyzed tumor changes in 25 patients who underwent MRI during the irradiation period forglioblastoma. Among these, 27% had GTVs during the RT period that deviated from the CTVs established before RT. Yang et al. [7] prospectively evaluated 11 cases of gliomas treated with IMRT (SIB). At the end of treatment, CTs and MRIs were performed and re-planned compared with the plan before treatment had started. They found that the size of the cavity and the GTV were significantly reduced, and the near-maximum dose to OARs and the irradiated volume of 10–50 Gy of the normal brain were significantly lower with re-planning. Champ et al. [6] compared the size of the GTV and the CTV on MRI after surgery at the time of RT planning in 24 cases of high-grade glioma, including glioblastoma. They reported that the initial GTV (tumor bed + T2/FLAIR high-intensity) and the initial CTV (GTV1 + 2 cm) were significantly reduced (mean 113.9 mL), and the boost CTV (boost GTV + 2 cm) was significantly increased (mean 32.5 mL). Stewart et al. [2] evaluated changes in the GTV and the CTV in 61 postoperative GBM patients who received 60 Gy in 30 fractions of RT using contrast-enhanced MRI at the time of treatment planning, at fractions 10 and 20 one month after completion. The GTV tended to decrease, but the number of cases deviating from the GTV at the time of treatment planning tended to increase over time. Bernchou et al. [13] investigated GTV displacement in 29 glioblastoma patients with MRI taken at 10, 20, and 30 fractions of RT and at 3 weeks after the end of r RT. The median distance of deviation from the original GTV was 5.7 (range 2.0–18.9) mm, 8.0 (range 2.0–27.4) mm, 8.0 (range 1.9– 27.3) mm, and 8.9 (range 1.9–34.4) mm at fractions 10, 20, 30, and follow-up. In recent years, treatment approaches using MRI linear accelerator (MRI Linac) have been reported. Mehta et al. [8] reported that daily imaging of three glioblastoma patients (two with total resection and one with other tumor and residual edema) using MRI Linac showed a gradual decrease in edema and tumor volume in all three patients.

To the best of our knowledge, this is the first study to prospectively assess the utility of ART exclusively for glioblastoma. In this study, the analysis based on the resection rate indicated that the target may expand in cases of partial excision and biopsy. The improvement in target coverage by adaptive boost plans was shown only in non-GTR cases. This could be explained by the greater impact of target expansion caused by residual tumor growth during the treatment period. In contrast, the improvement in CI and normal brain dose was shown only in GTR cases, which could be explained by the shrinkage of the resection cavity during the treatment period. Based on the results of this study, it may be possible to change the ART method according to the resection rate (if the patient has difficulty with frequent MRI due to restlessness, claustrophobia, etc., and in a total resection case, we will omit MRImid and plan adaptive boost directly on CTmid).

Although the dose to the normal brain could be reduced by ART, there was no significant difference between the fixed boost and adaptive boost plan doses to the optic nerve, brainstem, and other OARs. This may be because the adaptive boost plan was planned so that the total dose to the optic nerve and brainstem would be within the tolerable dose when combined with the initial plan. Thus, it is necessary to pay attention to the total dose to the OARs when ART is performed.

The margin for RT for glioblastomavaries according to the institution of administration and its guidelines [14, 15]. At our institution, a CTV margin of 1 cm is used in daily clinical practice, and also in this study. This considerably small margin setting policy has been suggested by our previous report, which analyzed the recurrence patterns ofglioblastoma, showing that the frequency of marginal recurrence was not particularly high [16]. Since changes in the GTV size are closely linked to the changes in the CTV and the PTV regardless of the margin, the margin setting did not largely affect the results of our study.

This study has several limitations. First, an increase in the enhancement area on the MRI during the treatment period may indicate pseudo-progression [17–19], which may lead to unnecessary expansion of the target. Pseudo-progression has not been mentioned in any of the previous studies [2, 6, 7] on target changes during glioblastoma treatment. Regarding the tumor growth in this study, the increase in the area of enhancement during treatment was clinically judged to be a recurrence as far as we could determine, but it was not clear because bevacizumab was introduced early in some cases. There were no cases of apparent radiation injury, and even if the target was expanded adaptively, there was little additional risk as long as the treatment plan was determined considering the tolerable dose to the brain. Further investigation is needed to distinguish between pseudo-progression and recurrence during RT. Second, this study was performed using 3DCRT alone and did not include IMRT. Seven patients who were judged to require IMRT　boost were excluded from our analysis. All of them showed a significant increase in the PTV on the MRImid, and would be more likely to benefit from ART, if IMRT was unavailable. Therefore, we believe that this exclusion did not overestimate but rather underestimated the results of our study, which showed the efficacy of ART. Currently, IMRT for brain tumors is prevalent, and the study of ART in IMRT is suggested. Third, the timing of ART was established only once at a point before reaching 40 Gy. Recently, it was possible to observe daily changes in the target volume using an MRI Linac. Further studies regarding the appropriate timing and frequency of ART application based on daily image analysis are warranted.

## Conclusions

The present study showed that boost planning using MRI during the treatment period in postoperative irradiation of glioblastoma improved target coverage and reduced normal brain dose. By analyzing the results according to the resection rate, we can expect a decrease in normal brain dose in patients withGTR and an increase in coverage in those with PR or biopsy. More appropriate timing of ART should be determined in the future studies.

## Data Availability

The datasets used and/or analysed during the current study are available from the corresponding author on reasonable request.
